# Systematic Identification and Analysis of Light-Responsive Circular RNA and Co-expression Networks in Lettuce (*Lactuca sativa*)

**DOI:** 10.1534/g3.120.401331

**Published:** 2020-07-23

**Authors:** Zhenchao Yang, Zhao Yang, Yingge Xie, Qi Liu, Yanhao Mei, Yongjun Wu

**Affiliations:** * College of Horticulture,; † College of Life Sciences, and; ‡ College of Science, Northwest A&F University, 712100 Yangling, Shaan Xi, China

**Keywords:** Light, Circular RNA (circRNA), co-expression network, microRNAs (miRNAs), lettuce

## Abstract

Circular RNA (circRNA) is a covalently-closed single-stranded RNA molecule that plays an important role in transcriptional regulation of gene expression in a variety of species. Light intensity is a pivotal environmental factor affecting plant growth and development. However, little is known regarding photoresponsive plant circRNAs. Here, we aimed to investigate the expression and function of circRNAs in lettuce leaves in response to different light intensity treatments. We performed RNA sequencing (RNA-Seq) on leaves of lettuce (*Lactuca sativa*) to determine circRNA expression profiles and reverse-transcription polymerase chain reaction (PCR) to validate the candidate circRNA molecules. We then combined bioinformatics approach to explore the function of the parental genes of circRNA, including network, Gene Ontology, and Kyoto Encyclopedia of Genes and Genomes pathway analysis. We identified 1650 circRNAs in lettuce, of which 1508 (86.40%) were derived from exons. Using real-time PCR, we characterized 10 validated differentially expressed circRNAs and their parental genes, all of which showed expression patterns consistent with RNA-Seq data. Interestingly, the expression of circRNA was, in some cases, inversely correlated with the expression of the parental gene. Furthermore, analysis of the circRNA–microRNA–mRNA network suggests that circRNAs may be involved in plant hormone signaling and chlorophyll metabolism during photoreactivity. These findings provide an essential reference basis for studying circRNAs’ biological mechanisms in light-treated plants.

There are two types of RNA in eukaryotic cells: coding, messenger RNA (mRNA), and non-coding RNA (ncRNA). Compared with mRNA, ncRNA accounts for the vast majority of the RNA world (Chen and Carmichael 2010). Different types of ncRNAs are found in cells, such as microRNAs (miRNAs), long non-coding RNAs (lncRNAs), and circular RNAs (circRNAs) (Memczak *et al.* 2013; Ye *et al.* 2015; Zuo *et al.* 2016). ncRNA has little or no protein-coding potential but plays a role in various biological processes (Wang *et al.* 2016). Non-coding transcripts have become increasingly important for a variety of plant functions, including healthy growth and development, as well as physiological and stress responses (Ariel *et al.* 2015; Wang *et al.* 2017a). With the development of high-throughput sequencing technology and efficient Big Data analysis, additional non-protein coding genes have been identified and characterized as effectors of plant responses to environmental stress. Indeed, non-protein coding genes play a crucial role in the stress response of wheat, including genes that give rise to fungal-reactive lncRNA (Zhang *et al.* 2016a) and dehydration-reactive miRNAs (Ma *et al.* 2015).

circRNA is an endogenous ncRNA that is single-stranded RNA generated by the head-to-tail joining of pre-mRNA (back-splicing) (Lasda and Parker 2014). The 5′- and 3′-termini of circRNA are joined together to form a covalent closed-loop structure (Ebbesen *et al.* 2017). The size of spliced circRNAs ranges from < 100 nt to > 4000 nt, but is usually only a few hundred nucleotides (Zhang *et al.* 2014; Lu *et al.* 2015). According to their genomic location, circRNAs are classed into exon, intron, intergenic, and exon-intron molecules (Chen 2016). According to preliminary studies, intron circRNA more commonly regulates its parental gene than exon circRNA (Wang *et al.* 2017b). Recently, Lu *et al.* (2015) reported the presence of 2354 circRNAs in rice and found that rice circRNAs negatively regulate their parental genes. CircRNAs appear to be differentially enriched in response to dehydration stress in wheat and foliar application of micronutrients (iron and zinc) in barley (Darbani *et al.* 2016; Wang *et al.* 2017c). Zuo *et al.* (2016) identified 854 circRNAs in tomato, of which 163 showed cold-response expression. These findings indicate that circRNAs are abundant in plants and may play important roles in response to abiotic stresses. Additional studies documented circRNAs’ role in Pi-starvation stress in rice, and reported the expression profiles of circRNAs in *Arabidopsis thaliana* in response to heat, and low light and high light stress (Ye *et al.* 2015; Pan *et al.* 2018). These reports indicate that circRNAs are widely involved in many biological processes, such as plant growth and development, and stress response.

Light is one of the most critical environmental factors. It is a source of energy and also a regulator of plant physiological adaptation (Cheng and Tu 2018). Typically, plant undergoes a series of adaptation steps to light intensity to maintain growth performance and health (Vialet-Chabrand *et al.* 2017). Comparative transcriptome analysis of tomato gene expression patterns under dynamic illumination revealed significant differences in gene expression under dynamic illumination and constant light conditions, with functional enrichment of plant-pathogen interactions, plant hormone signal transduction, metabolite production, and photosynthesis (Delprato *et al.* 2015; Felemban *et al.* 2019; Ding *et al.* 2019). Viršilė *et al.* (2019) compared the effects of different light intensities (100–500 μmol⋅m^–2^⋅s^–1^) and photoperiods (12–24 h) on the growth and nitrate assimilation of red and green lettuce (*Lactuca sativa* L.). After an abnormally long lack of light, plant responds to changes in light that alter the biogenesis of miRNAs (Achkar *et al.* 2018). Li *et al.* (2017a) used high-throughput sequencing to identify peach (*Prunus persica*) miRNAs that responded to UVB radiation in the greenhouse and showed that UV-responsive miRNAs are primarily involved in carbohydrate metabolism and signal transduction. Besides, Yang *et al.* (2019) systematically identified lncRNA during light-induced accumulation of anthocyanins in apple fruit and investigated the potential role of lncRNA in anthocyanin biosynthesis. Although circRNAs play an essential regulatory role in gene expression, the detailed function of most circRNAs remains unknown. In particular, little research has been done on the light response of circRNA in plant.

circRNA can inhibit the function of miRNA by acting as a miRNA sponge or bait in animals. For example, circRNA ciRS-7 (also known as CDR1as) contains more than 70 conventional miR-7–binding sites and can increase the expression of miR-7 target genes by strongly inhibiting human miR-7 activity (Memczak *et al.* 2013). However, there is no evidence that plant circRNAs function as miRNA sponges (Lu *et al.* 2015; Li *et al.* 2017b). Nevertheless, circRNA seems to interact and regulate miRNA, and mRNA at the transcriptional and post-transcriptional levels in most organisms. This mode of action is vital for exploring the potential role of plant circRNA in transcriptional and post-transcriptional regulation. The large-scale portal CircNet (Liu *et al.* 2016) has been developed to reveal the regulatory role of circRNA in circRNA–miRNA–gene regulatory networks.

Lettuce (*L. sativa* L., 2n = 18) is an important annual plant from the Compositae family and a substantial vegetable crop variety (Zdravković *et al.* 2014). Light plays a vital role in the growth and development of lettuce. Based on white light, high color rendering accelerates the growth of lettuce (Han *et al.* 2017). To date, no reports on the photo-responsiveness of lettuce circRNA have been published. The rapid development of RNA sequencing (RNA-Seq) technology resulted in the release of transcriptome datasets and lettuce reference genomes. A large number of transcriptome sequences (Reyes-Chin-Wo *et al.* 2017; Verwaaijen *et al.* 2018) provides useful sequence resources for the identification and systematic characterization of circRNAs in lettuce. We here aimed to explore the expression mode of circRNAs in lettuce, particularly, to investigate the light-responsive circRNAs and their potential regulatory effects in photoreactivity. We first identified and characterized circRNAs in high-throughput sequencing datasets of lettuce under different light intensity treatments and then used a series of bioinformatics methods characterize differentially expressed circRNAs, as well as functional annotations of light-responsive circRNA-parental genes. We show that lettuce circRNA involved in the response of light stimuli. These observations provide some reference value for the study of plant circRNA under different light intensity conditions.

## Materials and methods

### Plant material and growth conditions

The Hong Kong Glass lettuce seeds (Qingxian Qingfeng Seed Industry Limited Company, Hebei Province, China) were soaked for 48 h, surface-disinfected by gently shaking with 75% ethanol for 30 s, then washed 5-10 times with sterile water, and vernalized at 4° for 3 d. The treated seeds were grown in plastic pots containing a matrix soil at 26°. The average photon flux density in LHP-250 artificial climate chamber (Shanghai Sanfa Scientific Instrument Limited Company, Hebei Province, China) of the planting layer was 20%, 60 ± 2 μmol·m^–2^·s^–1^ (Las_WL group. low light intensity); 60%, 175 ± 2 μmol·m^–2^·s^–1^ (Las_ML group, medium light intensity); and 100%, 340 ± 2 μmol·m^–2^·s^–1^ (Las_SL group, high light intensity). The photoperiod was 14 h/10 h (light/dark) and humidity was set to 75%. Leaf tissue from 24-d-old lettuce was collected during the light, and all leaves of the co-axial unit of the leaf tip were removed. A single sample of lettuce leaves was cut and mixed (three biological replicates per set), wrapped in tin foil (2 g per sample), frozen in liquid nitrogen, and stored at –80° before further analysis.

### CircRNA library construction and sequencing

Total RNA was isolated and purified using TRIzol LS Reagent (Invitrogen 10296-010, Carlsbad, CA), following the manufacturer’s instructions. Approximately 5 μg of total RNA was used to deplete rRNA, according to manufacturer’s instructions of the Ribo-Zero rRNA Removal kit (Illumina, San Diego, CA). The obtained rRNA-depleted RNA was subjected to RNaseR (Epicenter, USA) treatment. The remaining RNA was fragmented into small pieces using Elution 2-Frag-Prime (ZYMO (R1015&1016)) at a 94° temperature, 8 min, ice bath 4 min. The cleaved RNA fragments were then reverse-transcribed with First-Strand Synthesis Mix Act D (ZYMO (R1015&1016)) using random primers (6-10bp) to generate cDNA. cDNA was then used as a template to synthesize U-labeled second strand DNA in a reaction with *Escherichia coli* DNA polymerase I, RNase H, and dUTP (TruSeq Stranded Total RNA HT Sample Prep Kit (Illumina RS-122-2203)). An A-base is then added to the blunt ends of each strand, preparing them for ligation to the indexed adapters. Each adapter contains a T-base overhang for ligating the adapter to the A-tailed fragmented DNA. Single-or dual-index adapters are ligated to the fragments, and size selection was performed with AMPureXP beads (Beckman A63881). For the final sequencing information from the first-strand cDNA, after the heat-unstable UDG enzyme processed the U-labeled second strand DNA, the ligation product was PCR amplified under the following conditions: initial denaturation at 95° for 3 min; 8 cycles of denaturation at 98° for 15 s, annealing at 60° for 15 s, and extension at 72° for 30 s; followed by a final extension at 72° for 5 min. The final insert size of the final cDNA library was 300-bp long (±50 bp), retaining the strand orientation of the RNA. Finally, paired-end sequencing was performed using Illumina Hiseq 4000 at LC Bio (Hangzhou, China), according to the protocol recommended by the supplier.

### Identification and differential expression of circRNA and parental genes

First, Cutadapt (Martin 2011) was used to remove the reads that contained adaptors, low-quality bases, and undetermined bases. Sequence quality was then verified by using FastQC (http://www.bioinformatics.babraham.ac.uk/projects/fastqc/). Bowtie2 (Langmead and Salzberg 2012) and Tophat2 (Kim *et al.* 2013) were used to map reads to the reference genome (GCA_002870075.1 Lsat_Salinas_v7, PRJNA432228, PRJNA173551). The remaining unmapped reads were mapped to genome using Tophat-fusion (Kim and Salzberg 2011). CIRCExplorer (Zhang *et al.* 2014; Liu *et al.* 2017) was used for *de novo* assembly of the mapped reads to circular RNAs. Then, back-spliced reads were identified in unmapped reads by using Tophat-fusion and CIRCexplorer. Unique circRNAs were identified in all samples. The differentially expressed circRNAs and Parental genes were selected based on log_2_(fold-change) > 1 or log_2_(fold-change) < -1, and statistical significance (*P* < 0.05) by using R package-edgeR (Robinson *et al.* 2010).

### Target gene prediction and functional enrichment analysis

To assess the potential function of circRNA, its parental mRNA was used for BLAST (https://blast.ncbi.nlm.nih.gov/Blast.cgi?PROGRAM=blastn&PAGE_TYPE=BlastSearch&LINK_LOC=blasthome) search analysis, and its function was classified according to the GO annotation (http://geneontology.org) and the KEGG pathway database (http://www.kegg.jp/kegg). circRNA enrichment analysis relative to the overall GO pattern and the KEGG pathway was performed using Blast2GO (Conesa *et al.* 2005) with Fisher’s exact test (FDR < 0.05). The GOseq method (Young *et al.* 2010), which is based on Wallenius non-central hyper-geometric distribution, was used for GO functional classifications to understand the distribution of gene functions at the macroscopic level. KEGG is the major public pathway-related database, and significantly enriched metabolic pathways or signal transduction pathways represented in the differentially expressed genes were identified by pathway enrichment analysis. For GO functional and pathway enrichment analysis, all differentially expressed genes were mapped to terms in the GO and KEGG databases, and significantly enriched GO and KEGG terms identified were compared to the genome background, with *P* < 0.05 as a threshold (Wu *et al.* 2006).

### Experimental validation of circRNAs and RT-qPCR of circRNAs and parental genes

Ten circRNAs were selected from the identified circRNAs based on the number of back-spliced sites and highly differential expression for experimental validation. Genomic DNA was extracted using the cetyltrimethylammonium bromide method (Murray and Thompson 1980), as a negative control of primers used for circRNA validation. circRNAs were validated in an aliquot of the total RNA sample (0.2 µg) used from RNA-Seq analysis. Prior to RT-qPCR, RNA samples were treated with DNase I (NEB, Beijing, China). Then, rRNA was removed using the Epicenter Ribo-Zero Gold kit (Illumina) and linear RNA was removed by incubation with 3 U⋅μg^–1^ RNase R (Epicenter) for 15 min at 37°. Two sets of primers for each circRNA were designed using Primer 5 program: an outward set expected to amplify only circRNA across the reverse-splicing junction (Table S2), and polymeric primers for the amplification of linear mRNA (Table S2).

The expression levels of circRNA and the parental gene were also quantified using a SYBR Fast qPCR Mix (Takara, Japan) and a BioRad CFX96 real-time PCR instrument (BioRad, USA). For the analysis, cDNA was synthesized using random primers with PrimeScript RT reagent Kit with gDNA Eraser (Takara, Japan), accordingly. The relative expression rate (∆Ct) of each circRNA was calculated using the 2^-∆∆Ct^ method (Livak and Schmittgen 2001). 18S rRNA was used as an internal standard control (Wu *et al.* 2016; Jeyaraj *et al.* 2017) and all reactions were repeated three times. The divergent and standard primers were designed for RT-qPCR using Primer 5, and were used to amplify circRNA and parental mRNA, respectively (Table S2).

### Co-expression network construction

Based on the alignment of circRNA sequences against miRBase version 21.0 (http://www.mirbase.org/) (Kozomara and Griffiths-Jones 2014), Targetscans (v7.0) (Lewis *et al.* 2005), miRanda [38] and CircNet (Liu *et al.* 2016) were used to predict the miRNA binding sites in lettuce circRNAs. miRBase compiles miRNA species from 34 plants. Based on the theoretically predicted interactions between circRNAs and conserved seed-matching sequences of miRNAs, the circRNA–miRNA–mRNA interaction network was visualized using Cytoscape 3.5.1 (Shannon *et al.* 2003). All the detected circRNAs and miRNAs were used for the interaction analysis.

### Data availability

Sequence data are available at NCBI and the accession number is GSE148578. The raw data are deposited in NCBI with SRA accessions numbers: SRP256288. Supplemental material available at figshare: https://doi.org/10.25387/g3.12196911.

## Results

### Identification and characterization of circRNAs in lettuce

To identify light-responsive circRNAs in lettuce at the genome-wide level, we produced three transcriptome datasets of light-treated lettuce, Las_SL (60 ± 2 μmol·m^–2^·s^–1^), Las_ML (175 ± 2 μmol·m^–2^·s^–1^), and Las_WL (340 ± 2 μmol·m^–2^·s^–1^). Three replicates of rRNA-depleted samples were used for each dataset. Approximately 1.02 billion original reads were obtained from the nine samples (Las_SL1, Las_SL2, Las_SL3, Las_ML1, Las_ML2, Las_ML3, Las_WL1, Las_WL2, and Las_WL3), equivalent to 150.32 G data (Table S1). After removing the adaptor and primer sequences, and short low-quality sequences, we obtained 811,624,664 clean reads. The Q20 and Q30 scores were both greater than 95% and the GC content was ≥45%, indicating high sequence quality (Table S1). Further, 92.38–93.86% of the resultant clean reads were successfully mapped to the lettuce reference genome (ftp://ftp.ncbi.nlm.nih.gov/genomes/all/GCF/002/870/075/GCF_002870075.1_Lsat_Salinas_v7/GCF_002870075.1_Lsat_Salinas_v7_genomic.fna.gz), with 626,043,281 reads uniquely mapped (Table 1). The corresponding 54,406,457 unmapped reads were retained for circRNA identification, with 5,481,756 candidate back-spliced junctions reads.

**Table 1:** Genome-wide identification of circRNAs in lettuce

Sample	Valid reads	Unique mapped reads	Unmapped reads	Candidate back-spliced junctions reads	Confident post reads	CircRNA number
Las_SL1	89132950	69762153 (78.27%)	5764358 (6.47%)	584034 (0.66%)	836	308
Las_SL2	87699358	68164847 (77.73%)	5531805 (6.31%)	538356 (0.61%)	990	321
Las_SL3	81245396	62978423 (77.52%)	5925249 (7.29%)	570611 (0.70%)	704	282
Las_ML1	112499652	87019476 (77.35%)	6906066 (6.14%)	717707 (0.64%)	1036	389
Las_ML2	98768636	74454209 (75.38%)	6082871 (6.16%)	578639 (0.59%)	863	320
Las_ML3	89441308	67692649 (75.68%)	6152748 (6.88%)	682352 (0.76%)	747	274
Las_WL1	86283614	68946078 (79.91%)	5985874 (6.94%)	791773 (0.92%)	398	185
Las_WL2	86368286	65637090 (76.00%)	6582693 (7.62%)	521422 (0.60%)	704	257
Las_WL3	80185464	61388356 (76.56%)	5474793 (6.83%)	496862 (0.62%)	558	198
All	811624664	626043281	54406457	5481756	6836	2534

Note: Las-SL, intensity light treatment group of lettuce (60 ± 2 μmol·m^–2^·s^–1^); Las-ML, medium light treatment group of lettuce (175 ± 2 μmol·m^–2^·s^–1^); Las-WL, weak light treatment group of lettuce (340 ± 2 μmol·m^–2^·s^–1^). Confident post reads, Number of reads corrected and filtered according to the sequence characteristics of the splice sites.

**Figure 1:**
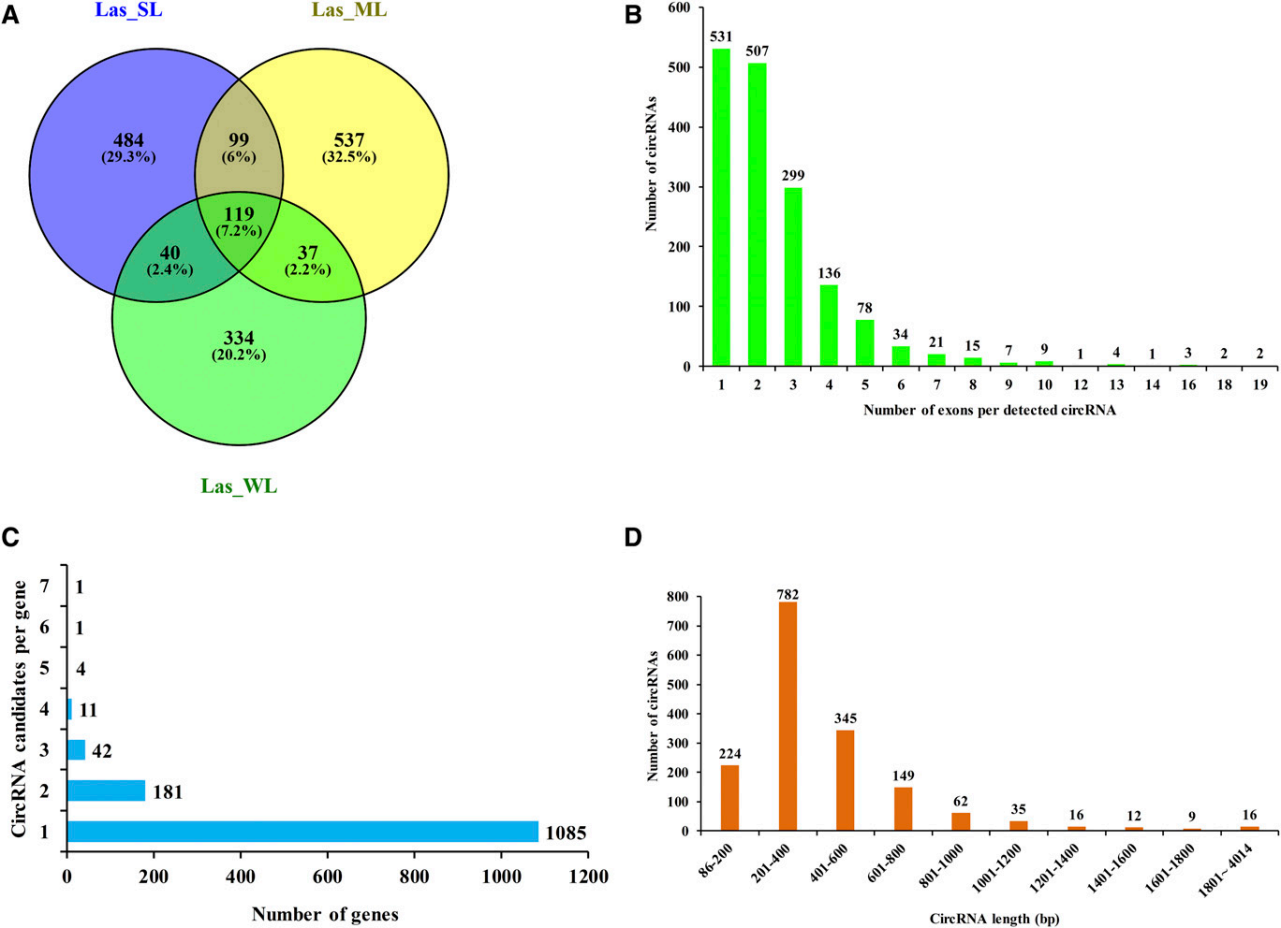
Genomic characteristics of lettuce circRNAs. (A) Venn diagram showing an overlap of the annotated circRNAs between the Las_SL, Las_ML, and Las_WL treatment groups. Las_SL, 340 ± 2 μmol·m^–2^·s^–1^ (high light intensity); Las_ML, 175 ± 2 μmol·m^–2^·s^–1^ (medium light intensity); Las_WL, 60 ± 2 μmol·m^–2^·s^–1^ (low light intensity). (B) Distribution of exons in detected circRNAs. (C) Distribution of circRNA readings observed in the dataset for each parental gene. (D) Distribution of circRNA length in lettuce.

Based on the positional relationship between circRNAs and the related genes, the back-spliced loci were aligned with the genomic region. Of these, 80.61% were located in exons, 15.59% in intergenic regions, and 3.80% in introns. Candidate circRNAs were identified based on the candidate back-spliced junctions in the rRNA-depleted RNA sequencing (RibominusSeq) dataset (Martin 2011). We used the CIRCexplorer2 program (Yin *et al.* 2017) to identify circRNAs. Consequently, 1650 circRNAs were identified in the three treatment groups, 742 in Las_SL, 792 in Las-ML, and 530 in Las_WL. Further, 119 circRNAs were shared among the three treatment groups, and 484, 537, and 334 circRNAs were specific to the treatment groups, accordingly (Figure 1A). The identified 1650 circRNAs were distributed among 754 scaffolds, with 1–2 circRNAs on 549 scaffolds (accounting for 72.81% of the scaffolds), and a portion of scaffolds containing > 2 circRNAs (Fig. S1). circRNAs can be produced from exons, introns, and intergenic regions (Chen 2016). In the current study, identification of total circRNAs based on the positional relationship between circRNAs and the related exons revealed the presence of only 1508 exon circRNAs. They were generated by exons of a single protein coding gene, and were the major circRNA type, accounting for 91.00% of 1650 circRNAs; 142 circRNAs were produced by introns. Approximately 89.27% of circRNAs harbored only 1–4 parental gene-derived exons (Figure 1B). The annotations of these circRNAs identified 1325 unique parental protein coding genes, indicating that some parental genes produced more than one circRNA, although most genes produced only one circRNA (Figure 1C).

These observations indicate that circRNA in lettuce are produced in different genomic regions via different splicing. Some genes produce more than one circRNA by alternative reverse-splicing or a combination of different exons/introns. In addition, most of the identified circRNAs were 86–800 bp in size (approximately 90.91% of all circRNAs). In particular, 201–400-bp circRNAs were the most numerous (Figure 1D).

### Analysis of differential expression patterns of circRNAs and the parental genes in lettuce

**Figure 2:**
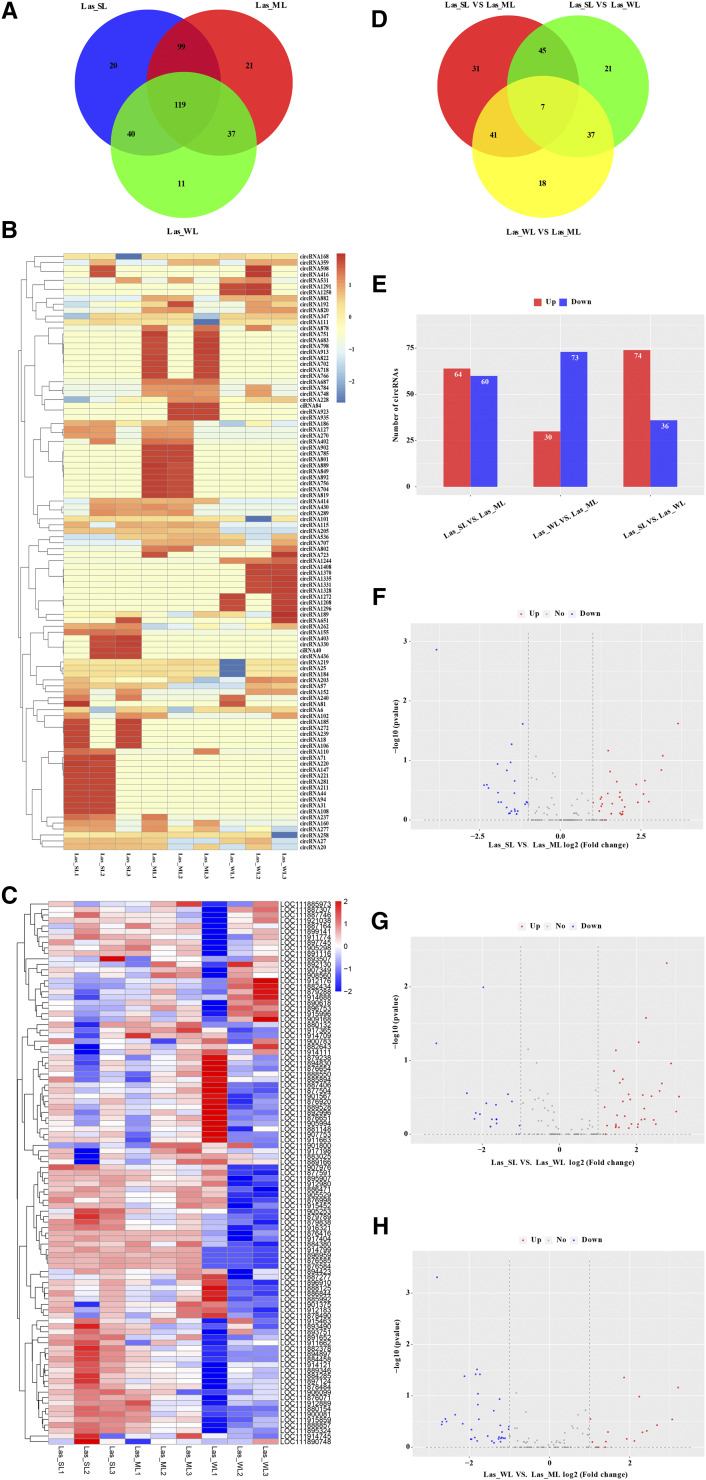
Differential expression patterns of circRNAs and parental genes in lettuce. (A) Venn diagram of the number of differentially expressed circRNAs between Las_SL, Las_ML, and Las_WL treatment groups. Las_SL, 340 ± 2 μmol·m^–2^·s^–1^ (high light intensity); Las_ML, 175 ± 2 μmol·m^–2^·s^–1^ (medium light intensity); Las_WL, 60 ± 2 μmol·m^–2^·s^–1^ (low light intensity). (B) Heat map of the expression pattern of differential circRNAs in Las_SL, Las_ML, and Las_WL treatment groups. The different colors indicate different gene expression. The color code for the Z-value (z = (x - µ)/σ, where x is the sample data, σ is the data standard deviation, and μ is the sample mean.) is shown on the right (blue to white to red, with the expression level from low to high, accordingly). (C) Heat map of the expression of different circRNA-parental genes in Las_SL, Las_ML, and Las_WL treatment groups. (D) Venn diagram of the number of differentially expressed circRNAs in pairwise comparisons of Las_SL, Las_ML, and Las_WL treatment groups. (E) A histogram of the differential expression of circRNAs in the processed pairwise comparison of lettuce samples. The number of up-regulated (red) and down-regulated (blue) circRNAs is shown at the top of each column. (F–H) Volcano plots of the overall distribution of differentially expressed circRNAs in pairwise sample comparisons (F) Las_SL *vs.* Las_ML, (G) Las_SL *vs.* Las_WL, (H) Las_WL *vs.* Las_ML. Abscissa, log_2_ (fold-change); ordinate, -log_10_ (p-value). The abscissa represents the differential expression (fold-change) of the gene, and the ordinate represents the statistical significance of the difference in the change in gene expression. Red, significantly differentially (p-values ≤ 0.01, T test) expressed gene that is up-regulated; blue, significantly differentially expressed gene that is down-regulated; gray, non-significantly differentially expressed gene.

We next determined the light-responsive circRNA expression in lettuce. We used R package (edgeR) (Robinson *et al.* 2010) to calculate the fragments per kilobase of transcripts per million mapped reads (FPKM) in lettuce samples from different light intensity treatments, counting reverse-spliced reads to quantify the circRNA expression. The expression values of all identified circRNAs were statistically distributed, and the expression levels and expression trends of genes between different samples were compared from the overall level (Fig. S2A, B). The gene expression pattern can be used as an indicator of its putative biological function. To determine which circRNAs were differentially expressed in the three treatment groups (three biological replicates per condition), circRNAs were filtered based on specific statistical thresholds [*P* ≤ 0.05 and | log_2_(fold-change) | ≥ 1]. Among the circRNAs identified in lettuce, 347 showed differential expression. In 347 circRNAs, only approximately 5.8% (20) Las_SL group circRNAs, 6.1% (21) Las_ML group circRNAs, and 3.2% (11) Las_WL group circRNAs were treatment-specific, while 34.2% (119) circRNAs were co-expressed in all treatment groups (Figure 2A). Hierarchical clustering analysis of circRNAs from the three groups revealed that circRNAs exhibited specific expression patterns in the different treatment groups (Figure 2B). Furthermore, the parental genes of the differentially expressed circRNAs exhibited similar specific expression patterns, up- or down-regulation, as that of circRNAs, except for a few parental genes that showed reverse expression patterns compared with those of their circRNAs (Figure 2C).

We also performed a pairwise comparative analysis of the differentially expressed circRNAs. The analysis revealed that seven circRNAs exhibited constitutive differential expression in the different treatment comparisons (Figure 2D). Further, 124 circRNAs were differentially expressed in the Las_SL *vs.* Las_ML comparison, the number of up-regulated is basically the same as down-regulated; 103 circRNAs were differentially expressed in the Las_WL *vs.* Las_ML comparison, the number of up-regulated is half of the down-regulated; and 110 circRNAs were differentially expressed in the Las_SL *vs.* Las_WL comparison, the number of up-regulated is twice that of the down-regulated (Figure 2E). These differentially expressed circRNAs may play specific roles in the light responsiveness in lettuce.

To visualize the overall distribution of the differentially expressed genes, volcano maps were prepared for circRNAs that were differentially expressed in each pairwise comparison. The number of up-regulated circRNAs in the Las_SL *vs.* Las_ML comparison (Figure 2F) and the Las_SL *vs.* Las_WL comparison (Figure 2G) was higher than the number of down-regulated circRNAs, while the number of circRNAs up-regulated in the Las_WL *vs.* Las_ML comparison (Figure 2H) occupied half of down-regulated. Collectively, the number of circRNAs up-regulated in the Las_SL and Las_ML groups was significantly (p-values ≤ 0.01, T test) greater than that in the Las_WL group. Because of the difference in growth conditions of lettuce associated with the different light intensity treatments, the expression of circRNA in different treatment groups appeared to be consistent with the characteristics of specific gene expression in the cell or tissue, or the developmental stage.

In addition, to explore the regulation patterns of differentially expressed circRNAs (in pairwise comparisons) under different treatment conditions, circRNAs were clustered according to the similarity of sample gene expression profiles. The heat map was then used to visualize differentially expressed circRNAs, demonstrating the expression of circRNA in different treatments (Fig. S2C–E). Biologically-relevant information obtained from the above heat map was consistent with those from volcano maps.

### Verification and analysis of circRNAs

**Figure 3:**
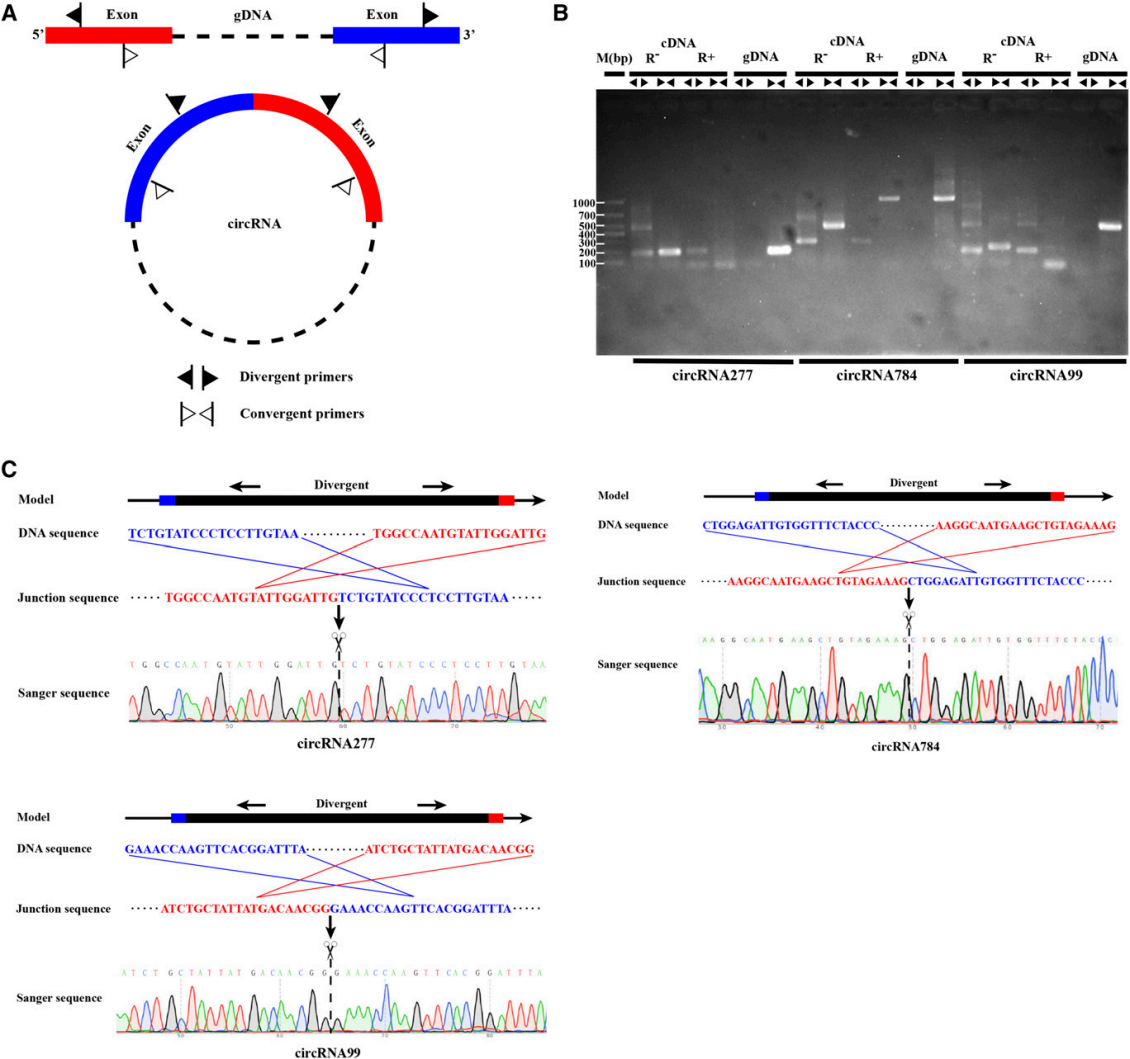
Verification of circRNA presence in lettuce. (A) A model of circRNA cyclization and convergence of convergent/divergent primers. (B) PCR analysis of three predicted circRNAs in genomic DNA and cDNA samples. Agarose gel electrophoresis revealed PCR products of the expected size. R^-^ indicates that no Rnase R-treated and R^+^ indicates that Rnase R-treated. (C) Detailed analysis of circRNA cyclization and Sanger sequence verification using divergent primers. The model of the parental gene structure is presented on top. The red and blue sequences indicate the 5′- and 3′-termini of linear mRNA, respectively, proposed to produce circRNA. The linker sequence and cyclization of circRNA are shown in the middle portion of each panel. Sanger sequencing data for a reaction involving divergent primers to amplify circRNA reverse-splicing linkages are shown at the bottom. The back-splice point from head-to-tail is indicated by scissors. The flanking sequences of the back-stitching are denoted in blue and red.

To confirm the identification of lettuce circRNAs, 10 circRNAs were chosen for experimental validation based on the number of back-spliced sites and highly differential expression. A set of divergent and convergent primers (Table S2) was designed for each circRNA, and used to amplify cDNA (including RNase R-treated reverse-transcript RNA) and genomic DNA. RNase R is typically selected for such analysis because while it digests all linear RNA, it does not digest lasso or circular RNA structures, thereby excluding noise signals associated with *trans*-splicing, genomic rearrangement, or potential PCR artifacts (Chen 2016). Theoretically, circRNA can be amplified using different primers from an RNA sample, but divergent primers cannot be used to amplify genomic DNA. By contrast, convergent primers amplify the linear form of the circRNA template in genomic DNA and RNA samples (Figure 3A). PCR amplification products were further analyzed by agarose gel electrophoresis and Sanger sequencing to confirm the occurrence of reverse-splicing. Indeed, the 10 analyzed circRNAs yielded a PCR product of the expected size, with a validated back-splicing point (Fig. S3). Three of these (designated circRNA277, circRNA784, and circRNA99) are shown in Figure 3B, C.

### Gene Ontology (GO) and Kyoto Encyclopedia of Genes and Genomes (KEGG) pathway analysis of differentially expressed circRNA-Parental genes

Since circRNAs exhibited a similar or opposite expression pattern to that of the parental protein-encoding genes, GO enrichment and KEGG pathway analysis of the circRNA-Parental genes were performed to explore the putative function of differentially expressed circRNA in lettuce. The Parental genes of 1283 differentially expressed circRNAs were divided into 728 functional terms. These were then divided into three main GO classification categories (biological processes, cell components, and molecule function), containing 379, 127, and 222 function terms, accordingly (Table S3).

**Figure 4:**
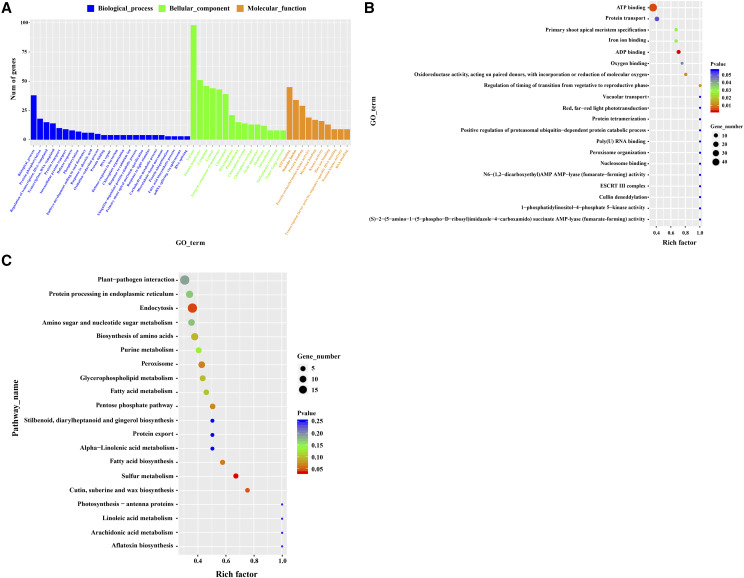
GO and KEGG enrichment analysis of differentially expressed circRNA-parental genes. (A) A histogram of GO enrichment analysis of differentially expressed circRNA-Parental genes. GO terms include biological processes, molecular functions, and cellular components. Abscissa, the GO annotation; ordinate, the number of genes. (B) A scatter plot of the GO enrichment analysis of differentially expressed circRNA-Parental genes. Abscissa, Rich factor. Rich factor indicates the ratio of the number of differential GO genes (S gene number) to the total number of GO genes (B gene number). The larger the Rich factor, the greater the GO enrichment. Ordinate, GO_Term (the GO function comment). In the scatter plot, the dot size represents the number of genes with a significant difference in the S gene number matched to a single GO. The dot color represents the p-value of the enrichment analysis, *i.e.*, the significance of enrichment; *P* ≤ 0.05 represents significant enrichment. (C) Scatter plot of KEGG enrichment analysis of differentially expressed circRNA-Parental genes. Abscissa, Rich factor. Rich factor indicates the ratio of the number of differential KEGG genes (S gene number) to the total number of KEGG genes (B gene number). The larger the Rich factor, the greater the KEGG enrichment. Ordinate, pathway term (the KEGG metabolic pathway). In the scatter plot, the dot size represents the number of genes with a significant difference in S gene number matching a single KEGG. The dot color represents the p-value of the enrichment analysis, *i.e.*, the significance of enrichment; *P* ≤ 0.05 represents significant enrichment.

For biological processes, the majority of circRNA-Parental genes were mainly involved in protein phosphorylation (GO: 0006468), regulation of transcription, DNA template (GO: 0006355), protein transport (GO: 0015031), defense response (GO: 0006952), and response to abscisic acid (GO: 0009737). Importantly, response to the light stimulus (GO: 0009416) was also a circRNA-rich term (Figure 4A; Fig. S4A–C). In the cell component class, in addition to the nucleus (GO: 0005634), plasma membrane (GO: 0005886), cytoplasm (GO: 0005737), and cytosol (GO: 0005829) circRNA-rich terms, we noted chloroplast (GO: 0009507) and chloroplast-related terms [*e.g.*, chloroplast matrix (GO: 0009570) and chloroplast envelope (GO: 0009941)] circRNA-rich terms. This suggests that the important roles of chloroplast-associated genes in the photoreaction in lettuce leaves are particularly sensitive to circRNA-related regulation. For molecular functions, enriched GO terms included ATP binding (GO: 0005524), molecular function (GO: 0003674), protein binding (GO: 0005515), protein serine/threonine kinase activity (GO: 0004674), metal ion binding (GO: 0046872), and kinase activity (GO: 0016301).

The GO enrichment analysis revealed that although the degree (Rich factor < 0.4) of enrichment of the term ATP binding (GO: 0005524) was not high, the number of genes that match the GO term with significant difference was high, and these genes are exceptionally significantly enriched (p-values ≤ 0.01, T-Test) (Figure 4B; Fig. S4D–F). Interestingly, we observed that the term red, far-red light phototransduction (GO:0009585) was significantly (p-values ≤ 0.05, T-Test) and highly (Rich factor = 1.0) enriched, but the number of genes with significant differences in this term was small. To further understand the biological function of the parental genes of 1283 differentially expressed circRNAs, in which the Parental genes of 734 differentially expressed circRNA were assigned to 86 KEGG pathways (Table S3). KEGG pathway analysis revealed that the parental genes of circRNAs are mainly involved in the metabolism of certain biomacromolecules, including fatty acid metabolism (ko01212), purine metabolism (ko00230), amino sugar and nucleotide sugar metabolism (ko00520), sulfur metabolism (ko00920), biosynthesis of amino acids (ko01230), fatty acid biosynthesis (ko00061), and pentose phosphate pathway (ko00030) (Figure 4C; Fig. S4G–I). In particular, the circRNA-Parental genes were significantly enriched (p-values ≤ 0.05, T-Test) in the endocytosis (ko04144) pathway and highly enriched (Rich factor = 1.0) in the photosynthesis-antenna proteins (ko00196) and plant hormone signal transduction (ko04075). Further, the plant-pathogen interaction pathway (ko04626) contained a large number of genes with significant differences in this pathway. This indicates that many of the parental genes of circRNAs are involved in protein synthesis and processing, photosynthesis, and response to stress.

### Validation of differentially expressed circRNAs and parental genes using reverse-transcription quantitative PCR (RT-qPCR)

**Figure 5:**
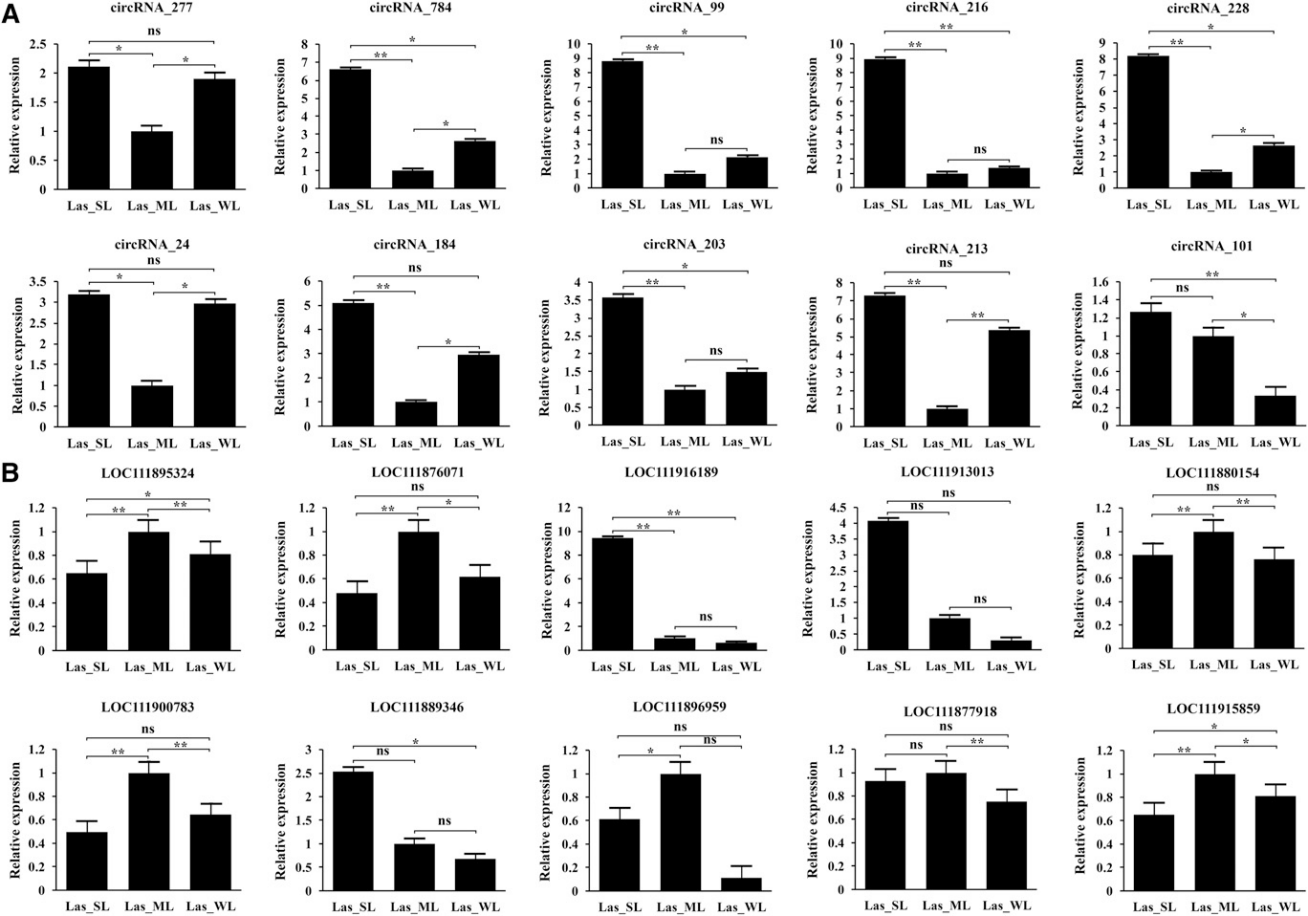
Expression of circRNAs and the parental genes in different treatment groups. (A) Differential expression of 10 validated circRNAs in the three treatment groups, as determined by RT-qPCR. (B) Differential expression of the parental genes of 10 verified circRNAs in the three treatment groups, as determined by RT-qPCR. In (A) and (B), the relative expression levels are shown on the y-axis. Values were represented as the mean ± SD. Error bars represent the standard deviations of three biological replicates. Diferences between the Las_SL *vs.* Las_ML, Las_ML *vs.* Las_WL and Las_SL *vs.* Las_WL were tested with a two-tailed *t*-test. *p-value ≤ 0.05, significant differences, **p-value ≤ 0.01, extremely significant differences. The positional relationship of the circRNA in (A) and its parental gene in (B) is corresponding.

To validate the differential expression patterns of circRNAs and the parental genes, we used RT-qPCR with divergent primers to analyze the abundance of 10 verified circRNAs (namely, circRNA277, circRNA784, circRNA99, circRNA216, circRNA228, circRNA24, circRNA184, circRNA203, circRNA213, and circRNA101) in different treatment groups (Table S2). The analysis revealed different expression patterns of these circRNAs in the different treatment groups. The RT-qPCR expression values of the selected circRNAs were consistent FPKM values calculated based on RNA-Seq, indicating that the RNA-Seq data were reliable (Figure 5A). We then used RT-qPCR to evaluate the expression of the corresponding parental genes of the selected circRNAs. The expression of six genes (the parental genes of circRNA277, circRNA784, circRNA228, circRNA24, circRNA203, and circRNA213) inversely correlated with the circRNA levels (Figure 5B). In addition, the expression of some parental genes was positively correlated with that of the corresponding circRNA. Hence, the relationship between circRNA abundance and the parental gene expression is not always straightforward, resulting in an important regulatory potential at the post-transcriptional level.

### Light-responsive circRNA–miRNA–mRNA co-expression network

**Figure 6:**
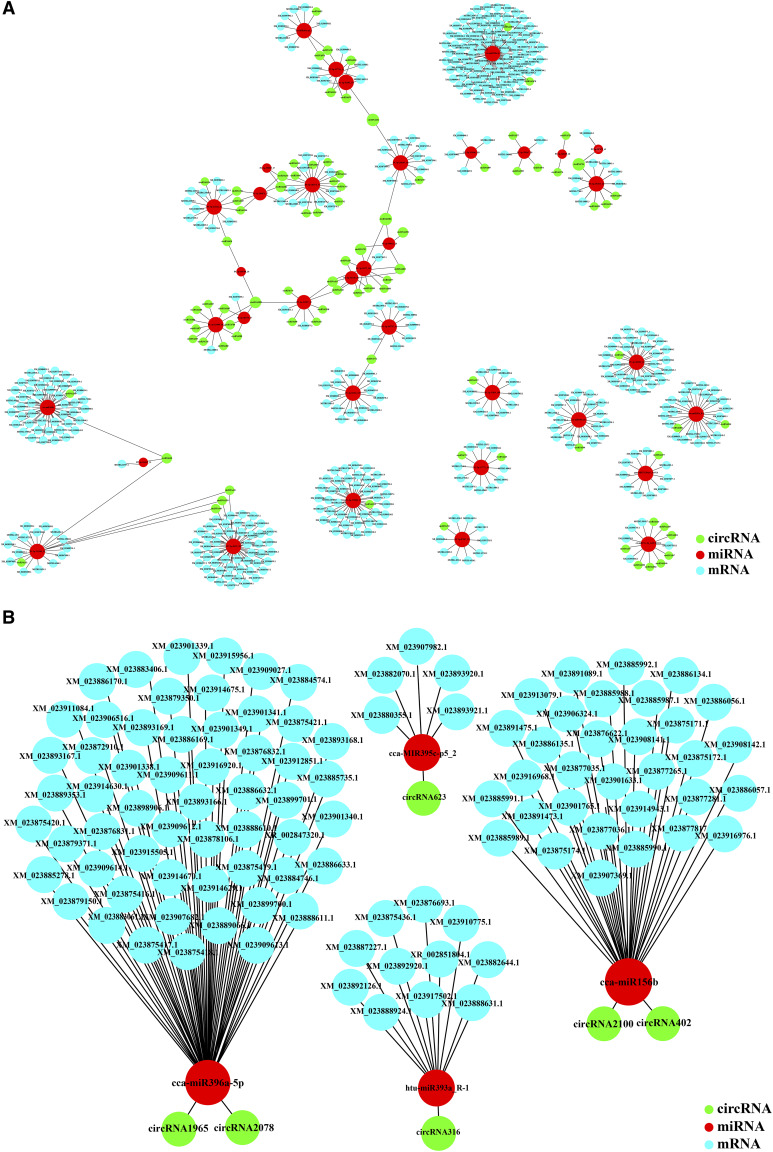
Potential interaction network of circRNA, mRNA, and miRNA in lettuce. The co-expression network is based on the interaction between circRNA–miRNA and miRNA–mRNA. (A) All identified potential circRNA–miRNA–mRNA co-expression networks. The green nodes represent circRNAs; the red nodes represent miRNAs; and the blue nodes represent mRNAs. (B) Potential light-responsive circRNA–miRNA–mRNA co-expression network.

To investigate whether circRNAs could target miRNAs and further influence the post-transcriptional regulation of gene expression in lettuce, the potential interactions between circRNA, miRNA, and mRNA were analyzed. We used Targetscans (v7.0) (Lewis *et al.* 2005), miRanda (Enright *et al.* 2003), and CircNet (Liu *et al.* 2016) determined potential interactions between 38 circRNA, 36 miRNAs, and 457 mRNAs, with the interacting circRNAs and mRNAs containing at least one predicted binding site for miRNA (Table S4). Considering the interactions between lettuce circRNAs and mRNAs, and certain conserved and newly identified miRNAs, the entire circRNA–miRNA–mRNA interaction network was reconstructed using Cytoscape (Figure 6A). The network contains 581 edges and 588 nodes, with multiple circRNAs (or miRNAs) predicted to interact with more than one miRNA (or circRNA). For example, we predicted that 12 circRNAs could target PC-3p-182575_51; three circRNAs could target PC-5p-214232_41 and PC-5p-284874_27; and other three circRNAs could target PC-5p-251306_33 and PC- 3p-40523_253. At the same time, four miRNAs are expected to bind to circRNA1992, while three miRNAs could be targeted by circRNA1986 (Figure 6A). In addition, six light-responsive circRNAs were analyzed, with respect to their interactions with 104 mRNAs and four miRNAs, to illustrate their potential linkages in response to light stress (Figure 6B). Functional annotations of the six circRNAs parent genes are mainly related to leaf development (GO:0048366), response to light stimulus (GO:0009416), chloroplast thylakoid membrane (GO:0009535), photosynthesis, light harvesting in photosystem I (GO:0009768), chloroplast envelope (GO: 0009941), chlorophyll binding (GO: 0016168), and pigment binding (GO: 0031409). Well-known miRNAs, such as cca-miR156b and cca-miR396a-5p, are also targeted by specific circRNAs in lettuce during the light response. Some of these circRNAs or miRNAs could play a key role in the circRNA–miRNA–mRNA interaction network.

## Discussion

To investigate the expression pattern and potential regulation of circRNA in lettuce under different light intensity treatment, we characterized the genome-wide circRNA of lettuce leaves by RNA-Seq. The presence and number of 1650 circRNA candidates were determined, and 10 differentially expressed circRNAs were validated using PCR method. Six of the validated circRNAs inversely correlated with parental gene expression levels, and these exceptions may be directly related to the mRNA abundance of the parental genes (Zhang *et al.* 2016b). The circRNA–miRNA–mRNA network indicates some key nodes, and multiple circRNAs can interact with a single miRNA, and vice versa. In addition, we found circRNAs are significantly enriched in chloroplasts-related GO terms and photosynthesis / response to light stimulation related KEGG pathways, suggesting that these circRNAs may participate in the regulation of their parental genes or interacted with mRNAs through co-expression network during leaf development or specific metabolites biosynthesis in lettuce.

Recent studies demonstrated that circRNAs are present in plants and play a role in plant responses to environmental stress (Ye *et al.* 2015; Zuo *et al.* 2016; Wang *et al.* 2017c; Liu *et al.* 2016). CircRNA is a unique type of RNA produced by a nonlinear reverse-splicing event between a downstream splice donor and an upstream splice acceptor. Recently, with the development of circRNA sequencing technology, a large number of circRNAs have been identified in plants and animals. These molecules respond to dehydration stress, nutrient elements, low and high temperature, and phosphate imbalance (Ye *et al.* 2015; Darbani *et al.* 2016; Wang *et al.* 2017c; Pan *et al.* 2018). The discovery of these widely expressed and highly conserved circRNAs increases the potential impact of ncRNA on cell function (Errichelli *et al.* 2017). Compared to animals, the biogenesis, regulation, and function of circRNA in plants are still relatively unclear (Zhou *et al.* 2017). Lettuce is an important horticultural crop that is highly sensitive to light intensity. To the best of our knowledge, to date, no studies have been published on the role of circRNA in light stress in lettuce.

In the current study, we conducted a genome-wide identification and characterization of the potential regulation of circRNA expression during light stress in lettuce. We detected 1650 circRNAs (Table 1; Table S1). Consistent with previous studies, these circRNAs were process-specific, with 484, 537, and 334 circRNAs specifically present in Lsa_SL, Lsa_ML, and Lsa_WL treatment groups, respectively. The number of circRNAs identified in lettuce was significantly lower than that identified in *Arabidopsis* (5861) (Chen *et al.* 2017), soybean (5372) (Zhao *et al.* 2017), maize (3715) (Tang *et al.* 2018), and rice (2354) (Lu *et al.* 2015), but higher than that in the tomato (854) (Zuo *et al.* 2016) and wheat (88) (Wang *et al.* 2017c). The differences reflect the different species used, study design (*e.g.*, experimental strategies or bioinformatics methods), tissues analyzed (*e.g.*, the leaf, root, fruit, stem, and shoot), and circRNA prediction tools used (*e.g.*, CIRI2, fnd_circ, and CIRCexplorer) (Yin *et al.* 2017).

Further, we used PCR with divergent primers to validate reverse-splicing of 10 circRNAs (Figure 3D, Table S2), confirming the reliability of the circRNA-seq data. We observed two non-specific amplification products of circRNA277 and circRNA99, verified by cDNA analysis after treatment with Rnase R. There are two possible explanations for this observation. First, PCR primers and reaction conditions may not have been applicable to the detection of all circRNAs. Second, detailed analysis of the detected circRNAs revealed that some Parental genes may produce more than one circRNA (by alternative splicing), consistent with previous reports for other plant species (Ye *et al.* 2015; Lu *et al.* 2015; Dong *et al.* 2016). Nevertheless, collectively, these observations indicate that high-throughput sequencing can be used for efficient and accurate identification of circRNA in lettuce. The circRNAs analysis in the current study suggests that circRNAs may form one of the smaller RNA families involved in the transcription in lettuce; however, they do expand the potential role of ncRNA in the complexity of cellular functions and regulatory processes in lettuce.

The response of plants to light stress is a very complex process involving many light-inducible genes and signal transduction pathways. In the current study, we explored the involvement of circRNAs in light response in lettuce. We found that 347 circRNAs were significantly differentially expressed under different light conditions (Figure 2B, G, I, K), that may be because the level of circRNAs varies with the specific treatment, *i.e.*, is process-specific circRNAs. RT-qPCR data for the selected circRNAs were consistent with the RNA-Seq data in that 9 out of 10 circRNAs were up-regulated in Lsa_SL and Lsa_WL samples, and down-regulated in Lsa_ML samples. This indicates that most circRNAs respond to light stress by up-regulation compared with their abundance upon exposure to normal light intensity. In *Arabidopsis*, heat stress induces increase of circRNAs numbers compared with the control conditions (Pan *et al.* 2018). In addition, circRNAs play different roles in plant biology, including negative regulation of parental genes (Lu *et al.* 2015; Zhao *et al.* 2017; Liu *et al.* 2017). The level of expression six Parental genes (circRNA277, circRNA784, circRNA228, circRNA24, circRNA203, and circRNA213 parental genes) was negatively correlated with the abundance of the corresponding circRNAs. As determined in the current study, during the growth and development of lettuce under different light intensity treatment, negative regulation of parental gene by circRNA is rare, indicating the possibility of competing regulation (Zhang *et al.* 2016b). The biological function of circRNAs may be consistent with the known function of the linear transcription of the parental gene (Ye *et al.* 2015; Lu *et al.* 2015). GO enrichment analysis of these differentially expressed parental genes indicated that the associated response to light stress, including GO terms, such as the response to stimulus [defense response (GO: 0006952) and response to light stimulus (GO: 0009416)], energy generation and conversion [ATP binding (GO: 0005524)], and ion transport [metal ion binding (GO: 0046872)] were specifically enriched (Figure 4; Table S3). In particular, chloroplast (GO: 0009507) and chloroplast-related terms [such as chloroplast matrix (GO: 0009570) and chloroplast envelope (GO: 0009941)], and plant hormone signal transduction (ko04075) may indicate an active regulatory role in lettuce photosynthesis. However, many metabolic pathways were also enriched, and the parental genes of circRNAs were mainly related to the metabolism of biomacromolecules. These circRNAs may be involved in the degradation of nutrients produced during the growth of lettuce leaf and redistributed to other parts of the plant. In addition, the parental genes of circRNA703 (LOC111884655) and circRNA96 (LOC111890907) may play important regulatory roles in the red, far-red light phototransduction (GO:0009585) pathway. Therefore, we believe that these circRNAs may compete with their parental genes and regulate the expression of their parental genes, which are valuable components of lettuce response to light stress.

Since circRNA can act as a sponge for miRNA, we analyzed mRNAs that share the same miRNAs as circRNAs to explore the function of circRNAs in the light response in lettuce (Figure 6; Table S4). In the circRNA–miRNA–mRNA co-expression network analysis, multiple circRNAs (or miRNAs) are predicted to interact with more than one miRNA (or circRNA). We were particularly interested in the networks of cca-miR156b and cca-miR396a-5p. These two miRNAs are involved in regulating plant growth, development, and stress (Wei *et al.* 2010; Cui *et al.* 2014; Chen *et al.* 2015). Under stress conditions, miR156 is induced to maintain the plant in the juvenile state for a relatively long period of time, whereas under permissive conditions, miR156 is suppressed to accelerate the developmental transition (Cui *et al.* 2014). In addition, Wei *et al.* (Wei *et al.* 2010) increased carotenoid levels in transgenic *Brassica napus* seeds overexpressing *Arabidopsis* miR156b. miR396s are a family of conserved microRNAs in plants that target the growth regulator family. miR396s interact with growth regulators to regulate plant growth, development, and stress tolerance. Chen *et al.* (2015) investigated the function of tomato miR396a-5p (Sp-miR396a-5p) in the response of Solanaceae to abiotic and biotic stresses, and found that the expression of Sp-miR396a-5p was down-regulated during pathogen-associated biotic stress. Functional annotation of light-responsive circRNAs revealed they are related cca-miR156b and cca-miR396a-5p, *e.g.*, leaf development (GO:0048366), response to light stimulus (GO:0009416), and photosynthesis, light harvesting in photosystem I (GO:0009768). These biological processes are related to the photoreaction process, further suggesting that circRNAs may exert potential regulatory effects on the photosynthetic growth stage in lettuce. In particular, chloroplast envelope (GO: 0009941), chlorophyll binding (GO:0016168), and pigment binding (GO:0031409) processes are involved in chlorophyll metabolism and promote plant growth. Collectively, the findings of the current study suggest that circRNAs play a role in the photosynthetic growth phase in lettuce by mediating chlorophyll metabolism and hormonal signaling pathways. However, given the current limitations of circRNA as a function of miRNA sponge in plants, we have only hypothesized the potential role while attempting to quantify their relative existence. Further exploration of the function of specific light-responsive circRNAs may have important constructive implications.

## Conclusion

We here identified 1650 circRNAs in the lettuce leaves exposed to light of different intensities, including 1508 (86.40%) exon circRNAs, and revealed differential circRNA accumulation during photoreaction. In addition, the expression of circRNA involved in the light response process was negatively correlated with the expression of the Parental genes, to certain extent. GO enrichment and KEGG pathway analysis of the parental genes with differentially expressed circRNAs indicated that response to light stimulus (GO:0009416), plant-pathogen interaction (ko04626), and plant hormone signal transduction (ko04075) may play an active regulatory role in lettuce light stress. Further, analysis of the circRNA–miRNA–mRNA network suggested that circRNA may be involved in chlorophyll metabolism and plant hormone signaling transduction process. These observations indicate that circRNAs may be important post-transcriptional regulators in the photoresponsive growth phase of lettuce leaf. This study further lays a theoretical foundation for exploring the regulation mechanism of specific light-responsive circRNA.
